# Subacute right ventricular pacemaker lead perforation: evaluation by echocardiography and cardiac CT

**DOI:** 10.1007/s12574-017-0337-5

**Published:** 2017-05-02

**Authors:** Reinier P. J. Boxma, Monique G. M. Kolff-Kamphuis, Ronald M. M. Gevers, Mohamed Boulaksil

**Affiliations:** 1 Department of Cardiology, Jeroen Bosch Hospital, Postbus 90153, 5200 ME ‘s Hertogenbosch, The Netherlands; 2 Department of Cardiology, Radboud University Medical Center, Nijmegen, The Netherlands

## Case

**Fig. 1:**
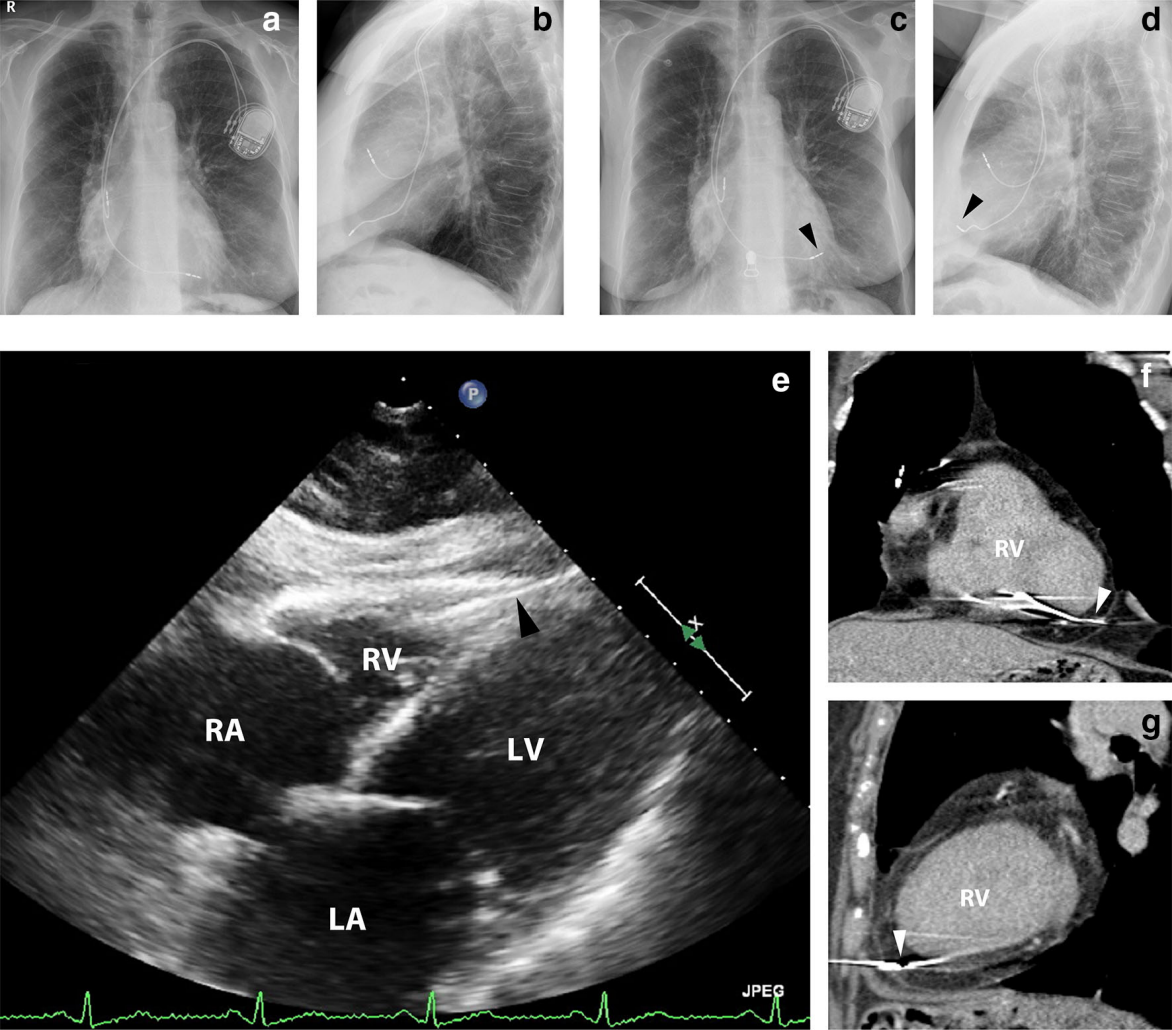
**a** Chest radiography [postero-anterior (PA) view] the day after pacemaker implantation. **b** Lateral view. **c** Chest radiography (PA view) upon presentation to the emergency room showing an altered position of the RV lead (*arrowhead*). **d** Lateral view (*arrowhead* shows altered lead position). **e** Transthoracic echocardiography (subcostal view) suggesting myocardial perforation of the RV lead through the RV apex (*arrowhead*), but without pericardial effusion, raising doubt as to whether the lead tip lay in the pericardial space. See also online video. *LA* left atrium, *LV* left ventricle, *RA* right atrium, *RV* right ventricle. **f** Thoracic computed tomography (coronal view) with lead tip clearly visible through the myocardium (*arrowhead*). *RV* right ventricle. **g** Thoracic computed tomography (sagittal view) showing lead tip running through myocardium (*arrowhead*). *RV* right ventricle

A 77-year-old female patient with a history of chronic obstructive pulmonary disease and paroxysmal atrial fibrillation presented to our emergency department with collapse and was diagnosed with sick sinus syndrome. Before pacemaker implantation, echocardiography showed normal dimensions of both atria and ventricles and normal systolic left and right ventricle (RV) function. A DDD-R pacemaker was successfully implanted using active-fixation leads without any acute complications. The day after implantation, sensing and pacing parameters were normal and unchanged. Chest radiography then showed a normal position of both leads (Fig. 1a, b). Subsequently, the patient was discharged the same day in good clinical health.

Four days later, however, the patient was readmitted because of sharp chest pain, unrelated to physical activity or posture. Pacemaker data showed a marked switch from bipolar to unipolar lead pacing and malcapture of the RV lead at maximal pacemaker output. Chest radiography revealed an altered RV lead position (Fig. 1c, d). Echocardiography suggested a perforation of the RV lead through the RV apex but without pericardial effusion (Fig. 1e and online video). This raised doubt as to whether the lead tip lay in the pericardial space. Ultimately, thoracic computed tomography (CT) showed that the lead went through the myocardium (Fig. 1f, g). The patient was subsequently transferred to a specialized pacing lead extraction center with surgical backup, where the RV lead was repositioned uneventfully.

## Discussion

Acute complications (<24 h) after pacemaker implantation occur in 3–7% of patients, of which ~1% is due to myocardial perforation (MP) [1]. In the subacute phase (1–30 days), MP may also occur, though less frequently, with an incidence of 0.03–0.4% of treated patients/year [2, 3].

Several predictors for development of MP are reported: old age (>80 years), female sex, RV apical lead positioning, and steroid use, while active fixation has been matter of debate [2, 3]. In our female patient, the RV lead was positioned in the RV apex. Although she was not over 80 years, she had nearly reached that age. In retrospect, the RV lead could have been positioned more septally.

MP has a wide variation in clinical presentation, ranging from clinically occult cases to cardiac arrest secondary to pericardial tamponade. Chest pain is the most frequently reported symptom [4].

Diagnosing MP may be challenging. Pacemaker parameter abnormalities are a first indication [4], though normal pacemaker function does not exclude this diagnosis [5].

Although routine chest radiography may be useful for evaluation of lead dislodgement, further imaging investigation is indicated if MP is suspected. Echocardiography can be useful in revealing presence of the RV lead in the pericardial space with or without pericardial effusion, but determining the exact lead tip position is difficult. CT, however, is superior in revealing lead tip position and in detecting MP (accuracy 92.9%, sensitivity 100%, and specificity 85.7%; echocardiography: 62.7%, 41.2%, and 84.2%, respectively) [4].

## Electronic supplementary material

Below is the link to the electronic supplementary material.

Online video: Transthoracic echocardiography (subcostal view) showing the RV lead through the RV apex. Pericardial effusion was absent. See also Fig. 1e. (AVI 7044 kb)

## References

[1] Ellenbogen KA, Wood MA, Shepard RK (2002). Delayed complications following pacemaker implantation. Pacing Clin Electrophysiol.

[2] Cano Ó, Andrés A, Alonso P, Osca J, Sancho-Tello MJ, Olagüe J, Martínez-Dolz L (2017). Incidence and predictors of clinically relevant cardiac perforation associated with systematic implantation of active-fixation pacing and defibrillation leads: a single-centre experience with over 3800 implanted leads. Europace..

[3] Sterlinski M, Przybylski A, Maciag A, Syska P, Pytkowski M, Lewandowski M, Kowalik I, Firek B, Kołsut P, Religa G, Kuśmierczyk M, Walczak F, Szwed H (2009). Subacute cardiac perforations associated with active fixation leads. Europace.

[4] Rajkumar CA, Claridge S, Jackson T, Behar J, Johnson J, Sohal M, Amraoui S, Nair A, Preston R, Gill J, Rajani R, Aldo Rinaldi C (2016). Diagnosis and management of iatrogenic cardiac perforation caused by pacemaker and defibrillator leads. Europace.

[5] Hirschl DA, Jain VR, Spindola-Franco H, Gross JN, Haramati LB (2007). Prevalence and characterization of asymptomatic pacemaker and ICD lead perforation on CT. Pacing Clin Electrophysiol.

